# Targeting MEST using cobicistat as a therapeutic strategy for gastric cancer through suppressing NF-κB signaling

**DOI:** 10.3389/fonc.2026.1901635

**Published:** 2026-08-04

**Authors:** Hongtai Cao, Huili Ye, Wentao Zhang, Weiwen Cai, Haobo Han, Junmin Zhu, Yuman Dong, Wengui Shi, Zhenming Zhang, Long Qin, Zuoyi Jiao

**Affiliations:** 1 Department of General Surgery, The Second Hospital & Clinical Medical School, Lanzhou University, Lanzhou, China; 2 Cuiying Biomedical Research Center, The Second Hospital & Clinical Medical School, Lanzhou University, Lanzhou, China; 3 Gansu Province High-Altitude High-Incidence Cancer Biobank, The Second Hospital & Clinical Medical School, Lanzhou University, Lanzhou, China; 4 Department of General Surgery, Hexi University & Zhangye People’s Hospital, Zhangye, China

**Keywords:** cobicistat, gastric cancer, MEST, NF-κB signaling, preclinical models, targeted therapy

## Abstract

**Background:**

Gastric cancer (GC) constitutes a substantial global public health challenge, and the lack of tractable molecular targets limits therapeutic progress. Mesoderm-Specific Transcript (MEST) has been implicated in tumor-related signaling, yet its functional role and druggability in GC remain undefined.

**Methods:**

The analyses of GC tissue microarrays and cohorts were performed to evaluate MEST expression and its clinical significance. CRISPR/Cas9-mediated MEST knockout was used to characterize its oncogenic functions in GC cells and xenograft models. Integrated RNA sequencing and pathway analysis was utilized to elucidate signaling pathways under the regulation of MEST. A structure-guided virtual screen combined with SPR binding and phenotypic assays were employed to discover small molecules targeting MEST. The therapeutic effects and mechanism of the lead compound were evaluated using GC cell lines, patient-derived organoids, cell-derived xenograft (CDX), and patient-derived xenograft (PDX) models.

**Methods:**

MEST expression in GC tissues was elevated and linked to poor prognosis. Functionally, genetic ablation of MEST impaired GC cell proliferation, invasion, migration, and suppressed tumor growth in CDX models. Screening of approved-compound libraries identified cobicistat as a previously unrecognized high-affinity candidate MEST-inhibitory compound. Cobicistat suppressed tumor growth across a panel of preclinical GC models, including cell lines, organoids, CDX and PDX. Mechanistically, MEST may drive GC progression by activating the NF-κB pathway, whereas cobicistat may antagonize MEST binding and blocked NF-κB pathway.

**Conclusion:**

MEST functions as a key oncoprotein driving GC progression via NF-κB activation. Cobicistat, a candidate MEST-inhibitory compound, exhibits favorable preclinical efficacy and safety, providing a promising candidate for targeted GC therapy.

## Graphical Abstract

**Figure:**
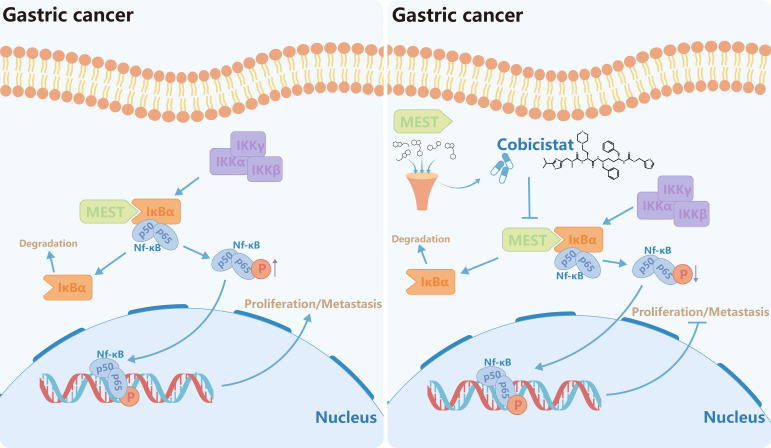
Targeting MEST with cobicistat inhibits GC growth via NF-κB signaling pathway. MEST overexpression drives GC progression by activating the NF-κB signaling pathway, whereas targeting MEST with cobicistat markedly suppresses tumor growth across multiple preclinical GC models.

## 1 Introduction

Gastric cancer (GC) is a widespread global malignancy and a leading cause of cancer deaths. Although great progress has been made in surgical resection, chemotherapy and targeted therapy, the clinical efficacy of patients with advanced GC is still unsatisfactory. Accordingly, it is imperative to explore the molecular mechanisms of GC progression and develop novel therapeutic targets (1, 2).

Dysregulation of imprinted genes has been increasingly recognized as a common event in tumorigenesis. Multiple imprinted gene products are involved in regulating cell proliferation, and the loss of imprinting of these genes may promote or inhibit tumor development (3, 4). The imprinted gene Mesoderm-Specific Transcript (MEST) is expressed exclusively from the paternal allele and resides at locus 7q32. It is frequently overexpressed in various embryonic and cancer tissues due to loss of imprinting, a phenomenon strongly associated with tumorigenesis (5–7). Previous studies have shown that MEST promotes epithelial-mesenchymal transition (EMT) by activating STAT3, which in turn induces Twist-1 nuclear translocation and facilitates metastasis in breast cancer (8). Similarly, in lung cancer, MEST exerts its oncogenic role by interacting with valosin-containing protein to coordinate the IκBα/NF-κB pathway. This interaction enhances IκBα breakdown, resulting in the release and nuclear trafficking of NF-κB dimers, thereby promoting tumor invasion and metastasis (9). Despite these advances, the molecular mechanisms through which MEST drives gastric carcinogenesis and the related targeted drug development remain poorly understood, highlighting the need for further investigation.

In this work, we explored MEST expression and its clinical relevance in GC. The results clearly reveals an oncogenic role for MEST in this malignancy. Downregulation of MEST inhibited GC cell malignant biological behavior. Mechanistically, MEST may drive GC development and progression by activating the IκBα-dependent NF-κB signaling. Importantly, we identified a candidate MEST-inhibitory compound, cobicistat, which exhibited anti-tumor effects against GC both in vitro and in vivo. Cobicistat exerts therapeutic efficacy against GC by targeting the MEST-NF-κB signaling axis. Our findings reveal that cobicistat, a candidate MEST-inhibitory compound, is a promising therapeutic candidate against GC.

## 2 Materials and methods

### 2.1 Patients and clinical specimens

The present retrospective cohort analysis was carried out among 165 participating patients who underwent gastrectomy for primary GC at the Second Hospital of Lanzhou University between 2013 and 2018. All patients had a confirmed diagnosis of primary GC. Human tissue specimens were collected after all participants provided signed written informed consent. All procedures of this investigation followed the tenets laid down by the Declaration of Helsinki.

### 2.2 Co-immunoprecipitation assay

A Co-IP assay was carried out using protein samples extracted from the AGS-MEST-OE3*FLAG stable cell line. Briefly, cells were recovered and fully washed with PBS. Protease inhibitors and immunoprecipitation (IP) lysis buffer were added to the cells, followed by centrifugation to collect the supernatant as the Input group sample. For the IP group, the supernatant was fully mixed with FLAG beads and incubated overnight at 4 °C. Subsequently, the beads were washed with IP lysis buffer, and the supernatant was discarded by placing the mixture on a magnetic separator. This washing step was repeated three times. Finally, SDS-PAGE sample loading buffer was added to the beads, and the mixture was boiled at 95 °C for 10 min in a dry bath incubator to denature proteins. The supernatant was collected using a magnetic separator and subjected to Western blot (WB) analysis.

### 2.3 Virtual screening for MEST inhibitors

The AlphaFold-predicted three-dimensional structure of MEST (UniProt ID: Q5EB52) was obtained from the UniProt database. Molecular docking pocket identification, virtual screening, and molecular docking simulations were performed using the commercial software MOE. The binding affinities of small molecules with MEST protein were evaluated by means of the Generalized-Born Volume Integral (GBVI) scoring function, which quantifies the binding free energy between the protein and individual small molecules. For molecular docking of small molecules to MEST, 500 random conformations of each small molecule were constructed and docked into the preselected binding pocket of MEST under rigid-receptor constraints. The conformation with the lowest predicted binding free energy was subsequently selected for further analysis and visualization.

### 2.4 Animal experiments

Female NSG immunocompromised mice aged six weeks were purchased from Model Organisms Center of Shanghai for this experiment. Fresh human GC tissues (**Supplementary Table 1**) or MKN-45 cells were inoculated subcutaneously in the mouse axillary region. Once the tumor volumes grew to 90 mm³, mice were split randomly into two treatment groups: one group received intraperitoneal injections of vehicle control, and the other group received 10 mg/kg cobicistat. During the treatment, drugs were administered every 2 days, and tumor volume and mouse body weight were measured simultaneously. Mice were euthanized when tumor volume approached 1500 mm³. All animal procedures in this work complied with globally acknowledged laboratory animal care criteria as well as the Guide for the Care and Use of Laboratory Animals, and complied with the 3R principles (Replacement, Reduction, and Refinement) for the ethical treatment of experimental animals. Mice received inhalation anesthesia with medical-grade isoflurane (purity ≥ 99.9%). With oxygen flowing at 1.0 L/min, 3.0% (volume fraction) isoflurane was delivered continuously through a mouse-specific nose cone for anesthesia induction. Experiments commenced after the loss of righting and pain reflexes as well as complete muscle relaxation. Humane euthanasia was performed using an overdose of inhaled isoflurane at the study endpoint.

### 2.5 Statistical analysis

All statistical calculations were performed using SPSS and GraphPad Prism software. All quantitative data were shown in the form of mean ± SEM. Two-group comparisons were achieved via two-tailed Student’s t-tests (paired or unpaired) based on experimental design. In vitro assays were replicated at least three times, utilizing both biological and technical replicates. Kaplan–Meier survival analysis was applied to evaluate patient survival outcomes, and log-rank tests were used to compare the differences between survival curves. A two-tailed P-value less than 0.05 was considered statistically significant (*p < 0.05, **p < 0.01, ***p < 0.001).

## 3 Results

### 3.1 MEST is overexpressed in GC and is a potential therapeutic target

**Figure 1:**
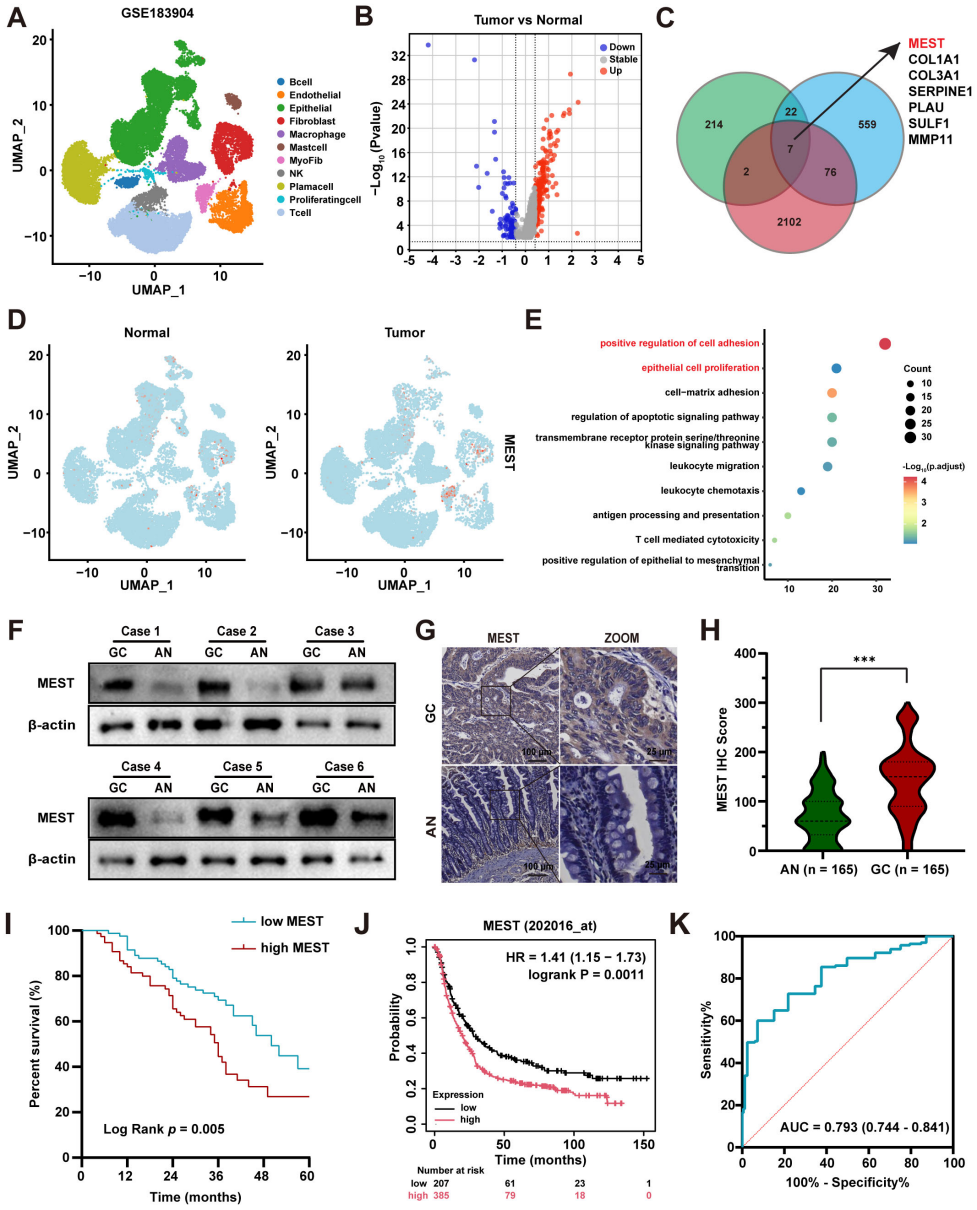
MEST is overexpressed in GC and is a potential therapeutic target. **(A)** The t-distributed stochastic neighbor embedding (t-SNE) plot showing clustering information from GSE183904. **(B)** Volcano plot of gene expression changes from GSE183904. **(C)** Venn diagram showing the overlap between the GSE183904 dataset, mRNA microarray and genes upregulated in GC, as derived from the TCGA database. MEST was detected in all 3 groups. **(D)** The t-SNE plots showing the MEST expression in GC tumor and normal tissues. **(E)** Gene Ontology (GO) analysis showing pathway terms for genes in GSE183904. **(F)** Immunoblotting of MEST in the tumor and corresponding adjacent normal tissues from GC patients (n = 6). **(G, H)** IHC staining of MEST and the quantification results in GC from 165 patients. **(I)** The correlation between MEST expression and survival in GC patients using clinical samples. **(J)** The correlation between MEST expression and survival in GC patients using a public Kaplan-Meier Plotter databases (http://kmplot.com/analysis/). Data are represented as mean ± SEM. **(K)** ROC curve analysis of the prognostic predictive value of MEST in GC patients using clinical samples. (***p < 0.001).

To explore promising new therapeutic targets against GC, we first analyzed single-cell RNA sequencing (scRNA-seq) data from GSE183904, and identified 245 upregulated genes (**Figures 1A, B**). Next, we performed mRNA microarray analysis to profile differentially expressed genes between tumor tissues and their adjacent normal counterparts from 16 GC patients, which revealed 664 genes that were upregulated in GC. We also retrieved a set of 2187 GC-upregulated genes from the TCGA database. Through intersection analysis of the datasets derived from these three sources, we identified 7 candidate genes (**Figure 1C**). Among these candidates, MEST was of particular interest, as its functional role in GC has been rarely investigated. To validate the screening results, we first analyzed scRNA-seq data from GSE183904 and observed that MEST expression was elevated in GC relative to non-tumor control tissues (**Figure 1D**). Gene Ontology (GO) enrichment was performed on differentially expressed genes derived from scRNA-seq datasets, we found that MEST exerted an influence on the cell adhesion and proliferation pathways (**Figure 1E**). Immunoblotting was performed to evaluate MEST protein expression in gastric cancer clinical samples, and elevated MEST expression was observed in carcinoma tissues compared with adjacent noncancerous tissues (**Figure 1F**). In addition, an immunohistochemistry (IHC) analysis was performed on a tissue microarray (TMA) containing 165 gastric cancerous and para-cancerous tissues, which indicated that MEST was indeed highly expressed in GC samples (**Figures 1G, H**). Then, prognostic analysis based on clinical specimens and public Kaplan-Meier Plotter data revealed that upregulated MEST protein expression predicted adverse prognosis for GC patients (**Figures 1I, J**). To assess MEST’s utility as a diagnostic biomarker, we performed ROC curve analysis using the clinical samples. The AUC for MEST was 0.793 (95% confidence interval [CI]: 0.744-0.841) in clinical samples (**Figure 1K**), indicating discriminatory power for distinguishing GC tissues from normal gastric mucosa. Collectively, the data establish MEST as a highly expressed oncogene in GC, with strong diagnostic and prognostic value. The high AUC and survival association highlight MEST as a promising candidate for use as a biomarker or therapeutic target.

### 3.2 MEST promotes GC growth in vitro and in vivo

**Figure 2:**
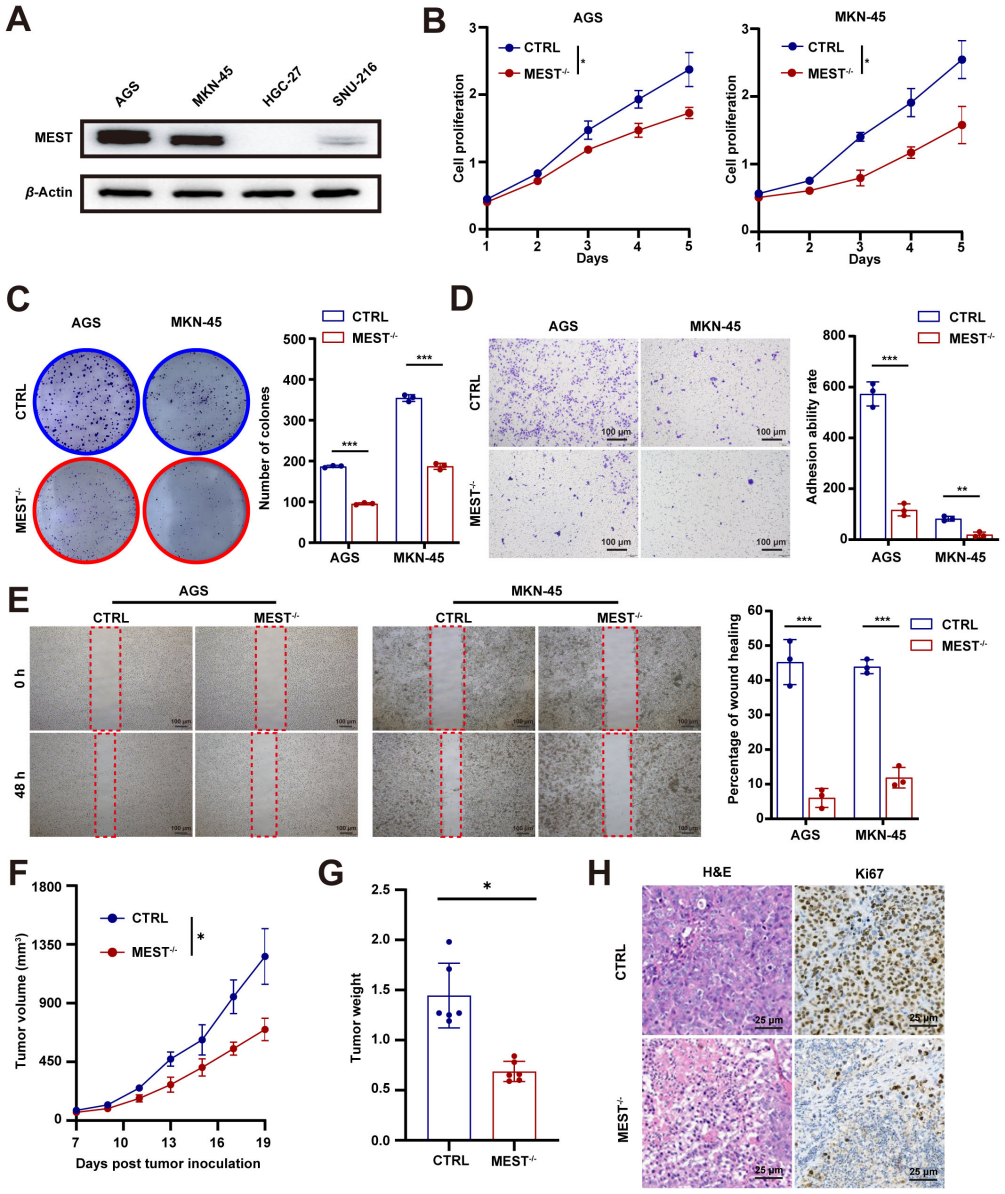
EST promotes GC malignancy in vitro and in vivo. **(A)** The protein expression of MEST in different GC cell lines (MKN-45, AGS, HGC27 and SNU216) was determined by Immunoblotting. **(B)** CCK-8 proliferation assays in MKN-45 and AGS cells from the control (CTRL) and MEST-/- groups. **(C)** Colony formation assays of AGS and MKN-45 cells from CTRL and MEST-/- groups. **(D, E)** The transwell and cell scratch assay was used to examine the metastatic abilities of MKN-45 and AGS cells. The percentage of migration and invasion rate (48h) in MEST-/- and CTRL groups (CTRL) after scratching and invasion. **(F–H)** Statistical tumor volume **(F)**, weight **(G)**, HE staining and immunohistochemical (IHC) analysis of Ki67 expression **(H)** in CTRL or MEST-/- MKN-45 cells-derived (n = 6). Data are represented as mean ± SEM. (*p < 0.05, **p < 0.01, ***p < 0.001).

Multiple GC cell lines were subjected to MEST expression detection for exploring the biological function of MEST in GC progression. Immunoblotting analysis demonstrated prominent MEST expression in AGS and MKN-45 GC cells compared with SNU216 and HGC27 cells (**Figure 2A**). Then, we applied the CRISPR/Cas9 technology to knock out the MEST gene in MKN-45 and AGS cells and assessed the impact of MEST depletion on the biological behaviors of GC cells. Cell viability and colony formation assays demonstrated that MEST deficiency suppressed proliferation of GC cells (**Figures 2B, C**). Furthermore, transwell and wound-healing tests indicated that MEST depletion impeded cell migration and invasion in GC cells (**Figures 2D, E**). To further verify the above results at the in vivo level, we established xenograft models using MKN-45 cells in NSG mice. The results confirmed that MEST knockout suppressed GC tumorigenesis and progression (**Figures 2F–H**). Collectively, these data indicate that MEST functions as an oncogene, promoting cancer growth and conferring tumorigenic potential in GC.

### 3.3 MEST promotes GC progression via the NF-κB pathway

**Figure 3:**
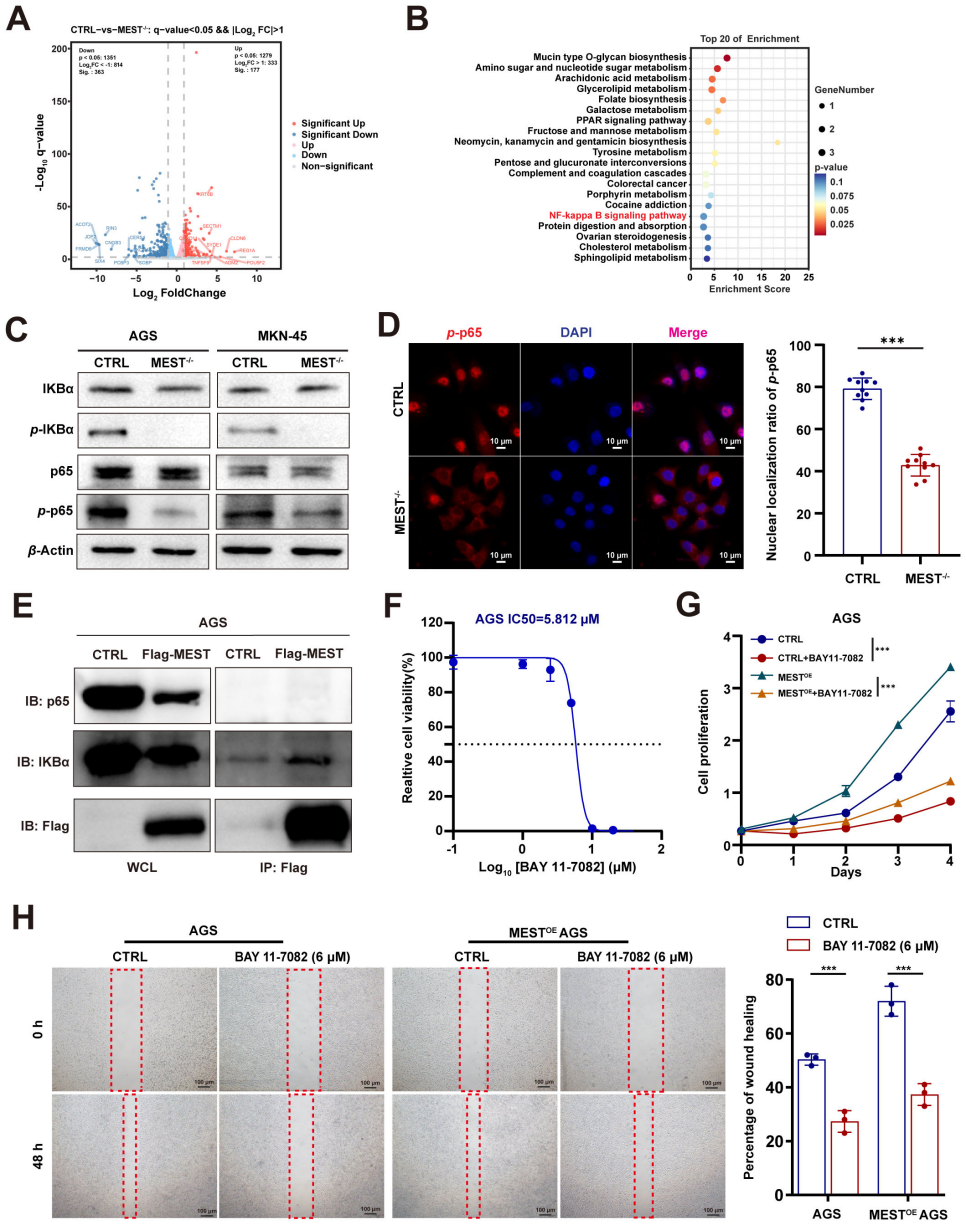
MEST Promotes GC progression through the NF-κB Pathway. **(A)** Volcano plot depicting the gene expression changes in the MEST knockout (MEST-/-) groups compared to control groups (CTRL). **(B)** Kyoto Encyclopedia of Genes and Genomes (KEGG) analysis showing pathway terms associated with the genes in the transcriptomic data. **(C)** Immunoblotting detected the effect of MEST-/- on NF-κB pathway-related markers. **(D)** Nuclear translocation analysis of phosphorylated p65 (*p*-p65) in CTRL and MEST-/- groups. Data are represented as mean ± SEM (***p < 0.001). **(E)** Semi-endogenous co-immunoprecipitation (Co-IP) assays showing MEST physically interacts with components of the NF-κB pathway. **(F)** Determination of BAY 11–7082 IC50 values in AGS cells. **(G)** CCK-8 assays detected the impact of BAY 11–7082 on the proliferation ability of AGS cells. **(H)** Migration assays showed that the effect of BAY 11–7082 on the metastasis of GC. (***p < 0.001).

To clarify how MEST exerts its oncogenic effects at the molecular level in GC, we conducted transcriptome sequencing in MEST-knockout AGS cells and wild-type cells. We screened 2630 differentially expressed genes (DEGs), consisting of 1279 upregulated and 1351 downregulated transcripts. (**Figure 3A**). Pathway enrichment was implemented for transcriptomic DEGs against the KEGG database, whereby we prioritized the top 20 statistically signaling pathways. Notably, among these pathways, MEST exerted the most influence on the NF-κB pathway (**Figure 3B**). Next, we performed immunoblotting and immunofluorescence assays to examine the effect of MEST on the NF-κB pathway. The results revealed that MEST knockout suppressed the expression level of p-IκBα and p-p65 in AGS and MKN-45 cells (**Figure 3C**). Moreover, nuclear translocation assays showed that MEST knockout inhibited the nuclear translocation of phosphorylated p65 (**Figure 3D**). We performed semi-endogenous Co-IP assays in AGS cells targeting MEST and core NF-κB components (p65, IκBα). Semi-endogenous Co-IP assays demonstrated that MEST was readily co-immunoprecipitated with endogenous IκBα in AGS cells, confirming their direct physical interaction (**Figure 3E**). We treated both MEST-overexpressing cells and parental AGS cells with the NF-κB inhibitor BAY 11–7082 and compared the phenotypic changes. The results showed that the half-maximal inhibitory concentration (IC_50_) of BAY 11–7082 was 5.812 μM (**Figure 3F**), and pharmacological inhibition of NF-κB by BAY 11–7082 recapitulated the core phenotypes observed in MEST knockout cells, including inhibition of cell proliferation (**Figure 3G**) and suppression of cell migration (**Figure 3H**). Collectively, these findings demonstrate that MEST may promote the occurrence and development of GC via the NF-κB signaling pathway.

### 3.4 Cobicistat was identified as a candidate MEST-inhibitory compound

**Figure 4:**
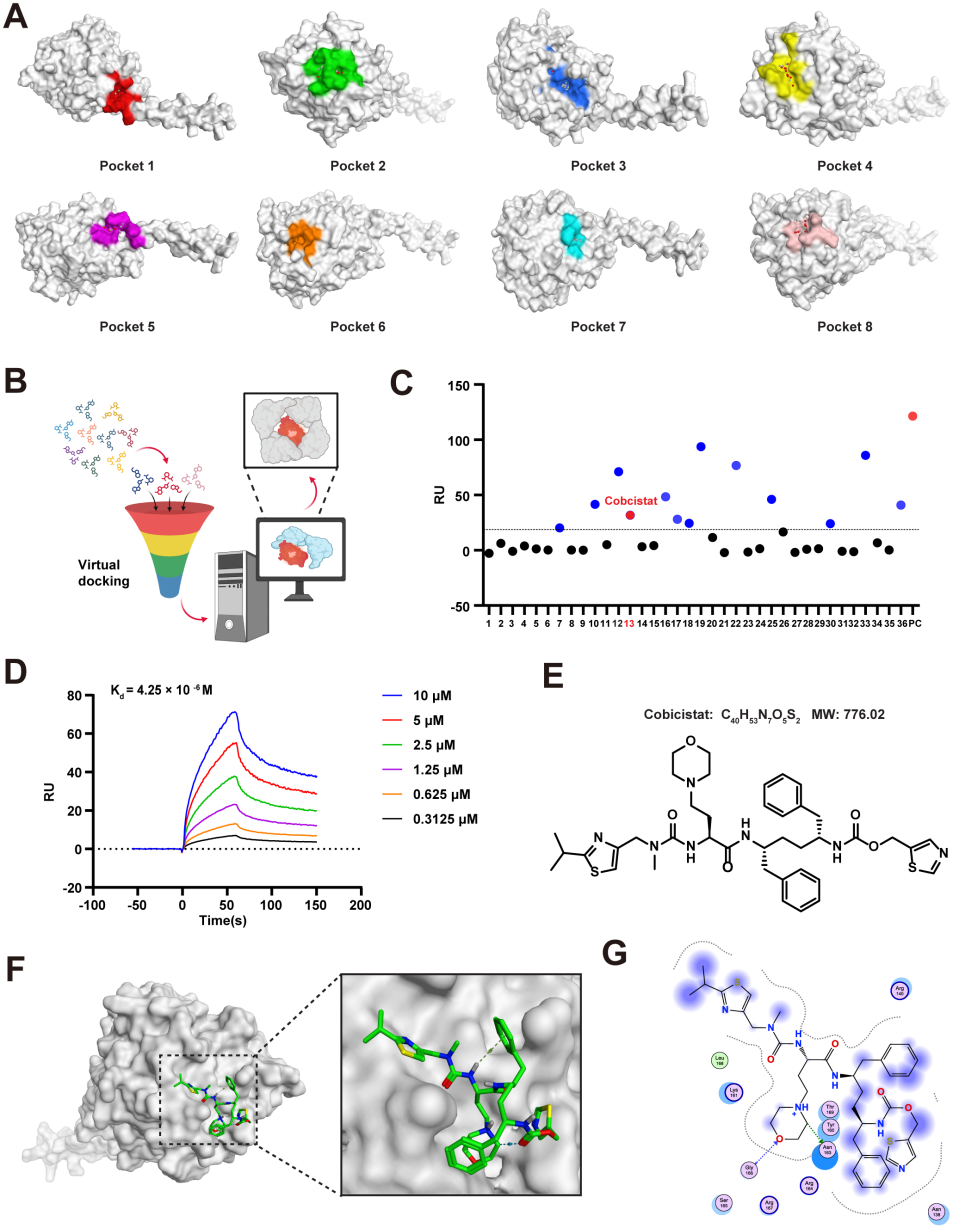
Cobicistat was identified as a novel MEST inhibitor. **(A)** Eight binding pockets of MEST were identified using the SiteFinder module of MOE software. **(B)** Virtual docking screening of MEST inhibitor from a library. **(C)** A preliminary screening of 36 compounds was performed by SPR. **(D)** Binding affinity of the cobicistat to human MEST assessed by SPR. **(E)** Molecular structure of cobicistat small molecule. **(F)** Computational model and the interaction of cobicistat with MEST complex. **(G)** The interacting amino acids and hydrogen bonds between the complexes.

To facilitate the development of targeted therapies for GC, we screened for small-molecule targeted drugs against MEST. Through in-depth analysis of the MEST protein structure model predicted by AlphaFold, eight distinct binding pockets of MEST were successfully identified using Site Finder (MOE, Version 2019) (**Figure 4A**; **Supplementary Table 2**). We then performed virtual screening of diverse chemical libraries containing 23,055 small molecules and identified 36 compounds that bind to pocket 8 of MEST (**Figure 4B**). These compounds were further subjected to screening via surface plasmon resonance (SPR) (**Figure 4C**; **Supplementary Table 3**). Ultimately, cobicistat was identified as a selective agent with high binding affinity for human MEST (KD = 4.25 × 10–6 M), as demonstrated by SPR analysis (**Figures 4D, E**). These results indicate that cobicistat exhibits a strong binding affinity for MEST, highlighting its potential as a candidate MEST-inhibitory compound. Further computational modeling was conducted to explore the interaction within the cobicistat-MEST complex (**Figure 4F**). The interaction forces within the complex and their corresponding amino acid residues are presented in **Figure 4G**.

### 3.5 Cobicistat suppressed GC malignancy

**Figure 5:**
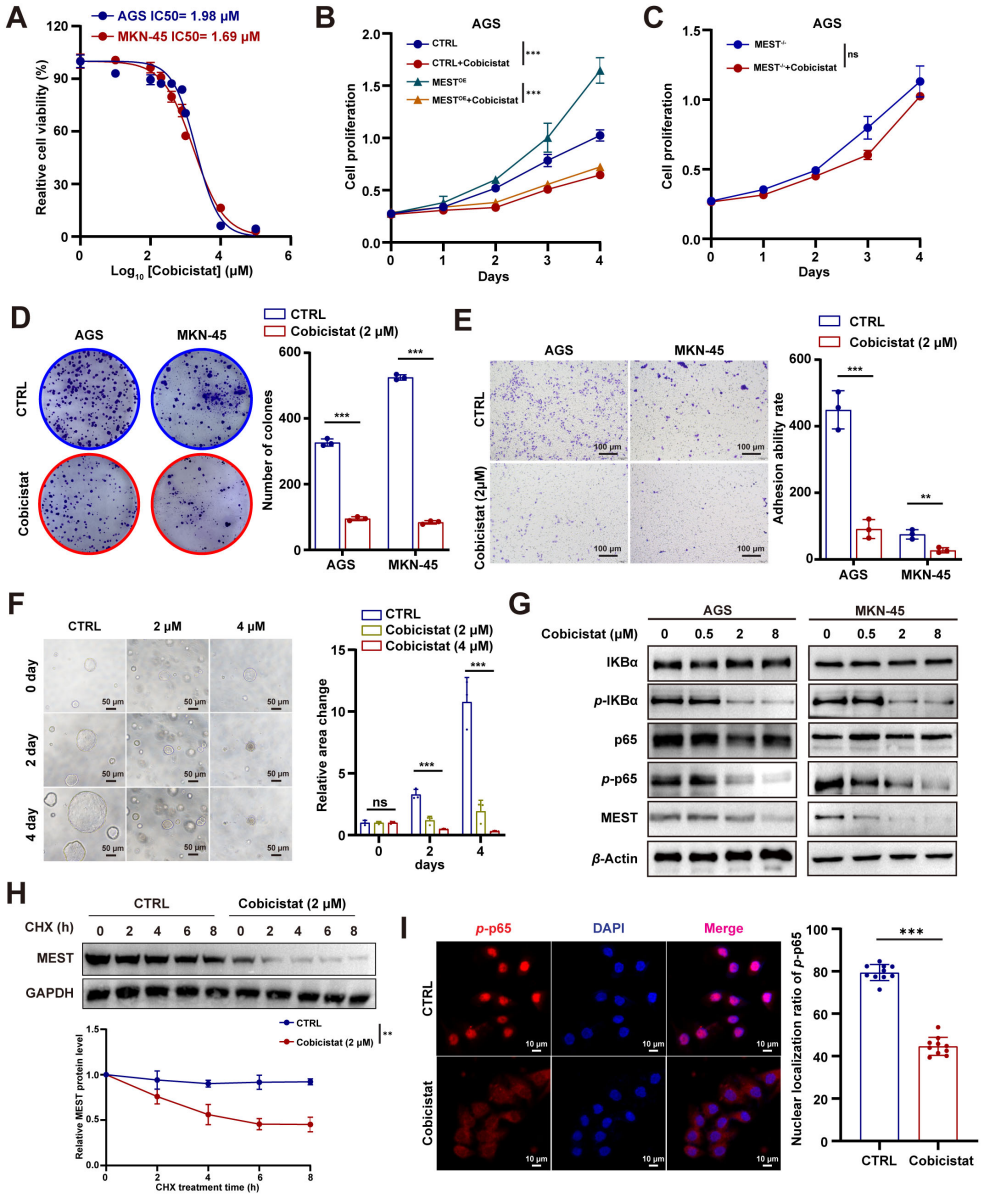
MEST inhibitor cobicistat inhibited GC malignancy. **(A)** Determination of cobicistat IC50 values in AGS and MKN-45 cells. **(B, C)** Cell proliferation of wild-type, MEST-/-, and MESTOE AGS cells treated with cobicistat was determined by the CCK-8 assay. **(D, E)** Colony formation and Invasion assays detected the impact of cobicistat on the proliferation and metastasis ability of AGS and MKN-45 cells. **(F)** The effect of cobicistat on one GC organoids at various concentrations. **(G)** Immunoblotting detected the effect of cobicistat on MEST and NF-κB pathway-related markers. **(H)** CHX chase assay demonstrating accelerated MEST protein degradation and shortened half-life upon cobicistat exposure versus control. **(I)** Nuclear translocation analysis of phosphorylated p65 (p-p65) in CTRL and cobicistat-treated groups. Data are represented as mean ± SEM. (**p < 0.01, ***p < 0.001).

To further examine the suppressive effect of the MEST inhibitor cobicistat on GC cells, we determined the IC_50_ values of cobicistat in AGS and MKN-45 cell lines, and the data indicated corresponding IC_50_ concentrations reached 1.98 μM and 1.69 μM (**Figure 5A**). We evaluated its effects on the proliferative behaviors of GC cells using CCK-8 assays. Cobicistat exhibited favorable efficacy in both MEST-overexpressing AGS cells and wild-type AGS cells, whereas its efficacy was negligible in MEST knockout AGS cells (**Figures 5B, C**). We subsequently assessed the suppressive influence of cobicistat on GC cells at a concentration of 2 μM using colony formation and invasion assays. The data revealed that cobicistat suppressed the proliferation and invasion of GC cells (**Figures 5D, E**). Furthermore, patient-originated GC tissues were utilized to establish an organoid system, followed by an investigation into concentration-dependent influences of cobicistat on organoid proliferation. The data demonstrated that cobicistat suppressed GC organoid growth at concentrations between 2 μM and 4 μM (**Figure 5F**).

To validate that cobicistat inhibits GC progression by targeting the MEST-mediated NF-κB pathway, we performed immunoblotting and quantitative real-time PCR (qPCR) assays. When the concentration of cobicistat reached 2-8 μM, the protein levels of MEST, p-p65, and p-IκBα were downregulated in AGS and MKN-45 cells compared with the control group (**Figure 5G**). The qPCR results demonstrated no significant differences in MEST mRNA levels in AGS and MKN-45 cells at any of the cobicistat concentrations tested (0, 0.5, 2, 8 μM) (Supplementary **Figure 1A**). We performed a cycloheximide (CHX) chase assay and found that cobicistat treatment significantly accelerates the degradation of MEST compared with the control group, resulting in a marked reduction in its protein half-life (**Figure 5H**). In addition, nuclear translocation assays revealed that cobicistat treatment inhibited p65 phosphorylation and concomitantly blocked its nuclear translocation (**Figure 5I**). Collectively, these findings suggest that cobicistat treatment accelerates MEST degradation, which in turn attenuates p65 phosphorylation and consequently blocks its nuclear translocation, culminating in the suppression of NF-κB signaling. Our data further underscore the potential of cobicistat as a promising candidate agent for gastric cancer therapy.

### 3.6 Cobicistat suppressed GC growth in a CDX model

**Figure 6:**
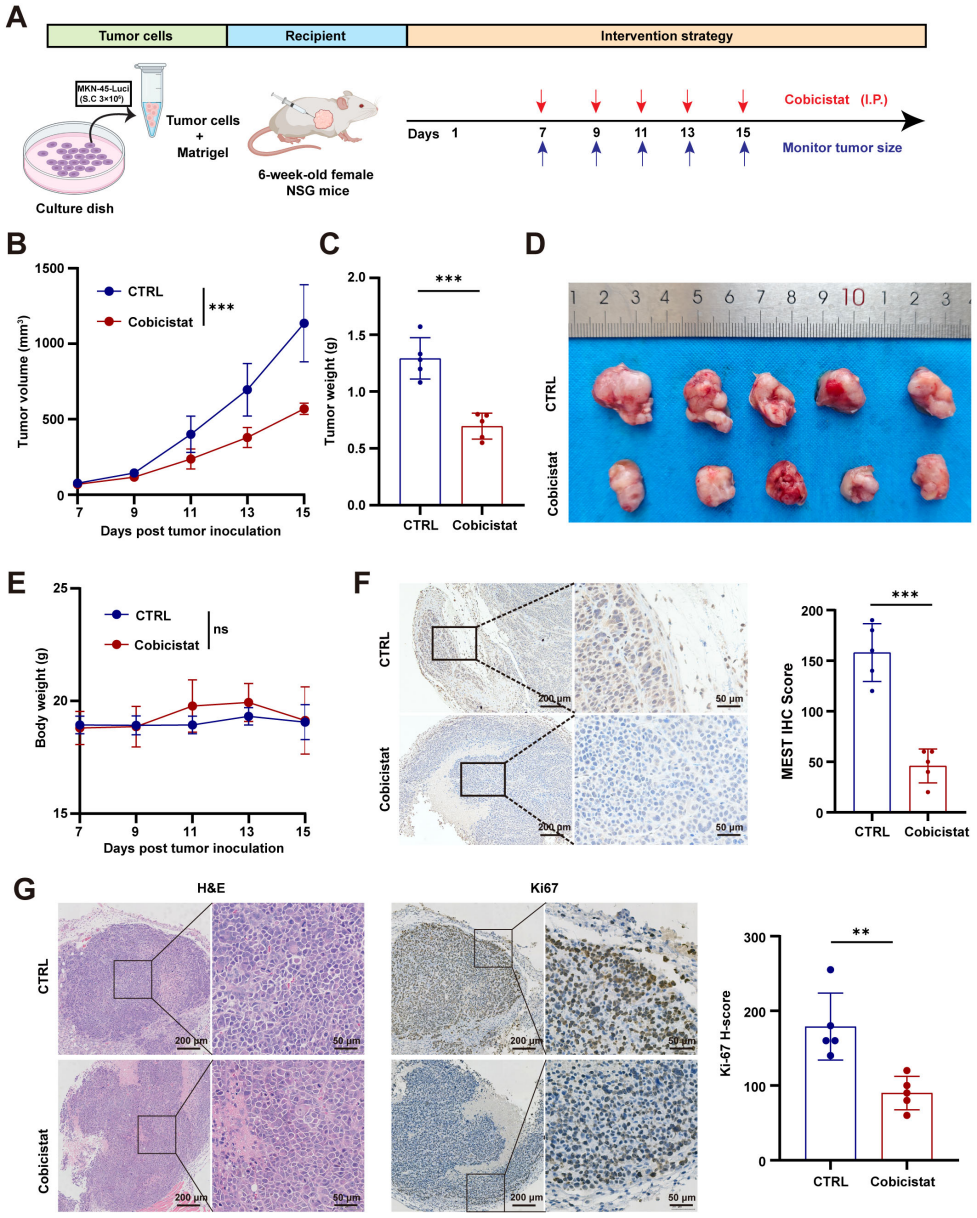
MEST inhibitor cobicistat inhibited GC growth in a CDX model. **(A)** The administration diagram of cobicistat in the CDX model. **(B–D)** Changes in tumor volume and weight in mice after cobicistat treatment. **(E)** Changes in body weight of mice after cobicistat treatment. **(F)** Changes in MEST expression in tumor immunohistochemical staining after cobicistat treatment. **(G)** Representative H&E and IHC staining of Ki67 in tumors of CDX mice treated with cobicistat. Data are represented as mean ± SEM. (**p < 0.01, ***p < 0.001).

Furthermore, we established a GC CDX model to examine the inhibitory effect of cobicistat in vivo. Once tumor volumes grew to 90 mm³, mice were treated with vehicle or the indicated concentrations of cobicistat via intraperitoneal injection at 10 mg/kg once every other day over a 15-day treatment period (**Figure 6A**). The results revealed that cobicistat suppressed tumor growth in the GC CDX model (**Figures 6B–D**), and no statistically meaningful body weight fluctuations could be found between either cohort of animals (**Figure 6E**). Furthermore, histological examination via H&E staining and IHC analysis of Ki-67 in xenograft tumors revealed that cobicistat inhibited MEST expression, concomitantly with a reduction in tumor cell proliferation (**Figures 6F, G**). Collectively, these results demonstrate the promising therapeutic potential of cobicistat against GC in vivo.

### 3.7 Cobicistat suppressed GC growth in a PDX model

**Figure 7:**
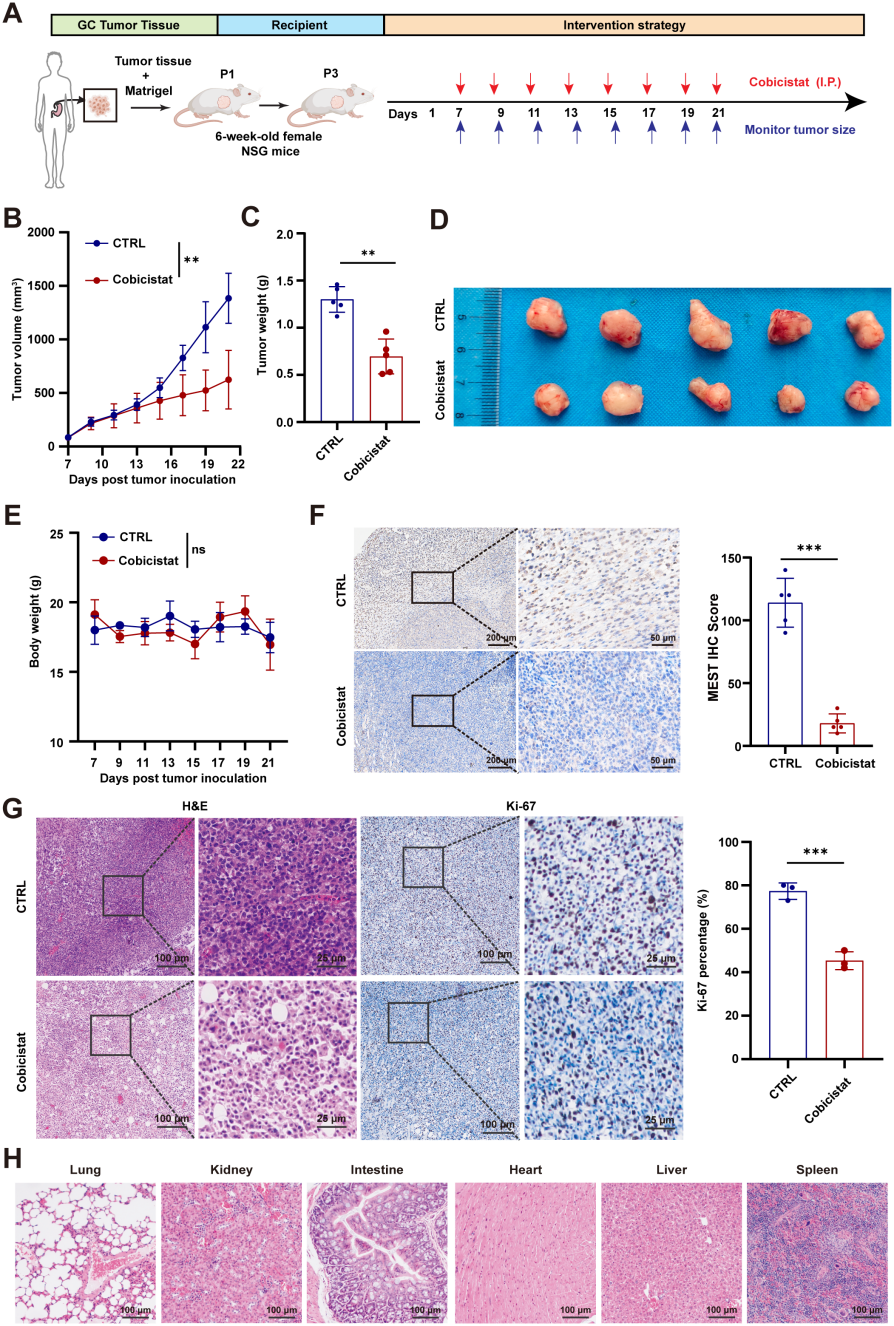
MEST inhibitor cobicistat inhibited GC growth in the PDX model. **(A)** The administration diagram of cobicistat in the PDX model. **(B–D)** Cobicistat significantly inhibited tumor volume, tumor weight and tumor image compared with the control group. **(E)** Changes in body weight of mice after cobicistat treatment. **(F)** Changes in MEST expression in tumor immunohistochemical staining after cobicistat treatment. **(G)** Representative H&E and IHC staining of Ki67 in tumors of PDX mice treated with cobicistat. **(H)** Representative H&E staining of various tissues from PDX mice treated with cobicistat. Data are represented as mean ± SEM. (**p < 0.01, ***p < 0.001).

To further elucidate the antitumor efficacy of cobicistat in vivo, we established a PDX model using tissues from a patient with GC (clinical characteristics are shown in **Supplementary Table 1**). Following tumor development up to nearly 90 mm³ in volume, mice were randomly assigned to receive either vehicle control or cobicistat (10 mg/kg) via intraperitoneal injection of blank solvent or cobicistat (10 mg/kg) every 48 h during three consecutive weeks (**Figure 7A**). The results showed that cobicistat restrained tumor progression within PDX xenograft models, as evidenced by reductions in tumor volume and weight (**Figures 7B–D**), and no statistically meaningful body weight fluctuations could be found between either cohort of animals (**Figure 7E**). Furthermore, H&E staining and IHC analysis demonstrated that cobicistat downregulated MEST expression and diminished the number of Ki-67-positive cells, indicating substantial suppression of tumor cell proliferation (**Figures 7F, G**). Moreover, cobicistat exhibited no significant adverse effects on normal tissues (**Figure 7H**). Collectively, these findings provide robust evidence for the therapeutic potential of cobicistat in suppressing GC growth in vivo and underscore its promise as a novel therapeutic strategy, warranting further preclinical and clinical development.

## 4 Discussion

Gastric cancer (GC) ranks among the deadliest human malignancies globally accompanied by elevated incidence and death rates, highlighting an urgent demand to decode complicated biological pathways governing tumor onset and advancement. MEST (alternatively designated PEG1) falls into the large α/β-hydrolase superfamily cluster (10). Extensive research has revealed that MEST expression is markedly upregulated in various cancers, including choriocarcinoma (11), breast cancer (8), lung adenocarcinoma (9, 12), and lymphoblastoid cancers (13). Intriguingly, our study extends these observations to GC, indicating a close association of MEST levels with unfavorable clinical outcomes among GC sufferers. Importantly, MEST knockout suppresses tumor growth, invasion and metastasis in both in vitro (cell lines and organoids) and in vivo (xenograft) models. This inhibitory effect is consistent with the previously reported oncogenic role of MEST in breast and lung cancer models, highlighting its conserved functional mechanism across different cancer types. Therefore, our findings establish MEST as a pivotal oncogene driving GC progression and highlight its broad therapeutic potential in GC.

NF-κB has been widely recognized as a critical regulator involved in multiple events linked to tumor progression, including cell proliferation, viability maintenance and metastatic spread (14). Constitutive activation of NF-κB is frequently detected in diverse human malignancies, while it is seldom present in normal tissues (15). In breast cancer, NF-κB has been shown to promote the progression of epithelial-mesenchymal transition (EMT) and endocrine therapy resistance (16, 17). Although the use of NF-κB-targeted inhibitors in cancer treatment is controversial, their combination with classical chemotherapy drugs has shown a synergistic effect (18, 19). Wang Y and coworkers revealed that MEST interacts with VCP to accelerate IκBα breakdown and initiate NF-κB signaling activation, which facilitates invasive and metastatic progression of lung carcinoma (9). Given the unique genetic background, microenvironmental characteristics, and clinical manifestations of GC, our group conducted further relevant studies on GC. Our data indicate that high MEST expression elevates the levels of p-IκBα and p-p65 and promotes their nuclear translocation, thereby driving the activation of NF-κB in GC. This process is significantly associated with GC proliferation, invasion, and metastasis. Rescue experiments targeting the NF-κB pathway validated the causal role of the MEST–NF-κB regulatory axis. These findings indicate that MEST exerts its oncogenic functions, at least in part, through the NF-κB signaling pathway. Consequently, developing inhibitors that target MEST, a cancer regulator of the NF-κB pathway, represents a promising therapeutic strategy for GC.

While the biological roles of MEST have been comprehensively investigated in breast malignancy and embryonic growth (8, 20), no therapeutic inhibitors targeting MEST in GC have been reported to date (21, 22). Here, we identified a candidate MEST-inhibitory compound, cobicistat, by screening the TargetMol and Selleck small-molecule compound libraries based on its catalytic site, ASN163. Our data showed that among the 13 candidate drugs screened via SPR, cobicistat exhibited the highest inhibitory efficiency against GC cell proliferation, with an IC50 of 1.69-1.98 μM. The antitumor efficacy of cobicistat was further evaluated across a spectrum of preclinical models, including cell lines, patient-derived organoids, cell-derived xenografts (CDX), and patient-derived xenografts (PDX). Mechanistically, we demonstrate that cobicistat targets MEST and suppresses NF-κB signaling, thereby contributing to the inhibition of gastric cancer progression. Nevertheless, we acknowledge that the number of PDX models evaluated in this study is limited, and further validation in larger and more diverse cohorts will be required to confirm the generalizability of our findings. Moreover, while our data support a role for NF-κB in mediating the oncogenic functions of MEST, additional downstream effectors or signaling cross-talk may also be involved. Therefore, we propose that MEST operates, at least in part, through NF-κB signaling in gastric cancer.

Cobicistat is a well-established CYP3A inhibitor, and accumulating evidence has suggested that its antitumor effects may be mediated, at least in part, through CYP3A inhibition or engagement with other off-target proteins (23). In the present study, we provide functional genetic evidence suggesting that MEST is a key mediator of the antiproliferative activity of cobicistat in the cellular context. Notably, in MEST-knockout cells, cobicistat treatment did not further reduce proliferation beyond the level already achieved by MEST ablation alone, with the proliferation of drug-treated knockout cells being statistically indistinguishable from that of untreated knockout cells. This findings indicate that the antiproliferative efficacy of cobicistat is largely dependent on MEST under the in vitro conditions, and do not support a major independent contribution from CYP3A or other alternative targets in this context. Nevertheless, given the abundant expression of CYP3A in the liver and intestine (24), and its potential to influence the tumor microenvironment through metabolism of endogenous substrates (25), we cannot formally exclude the possibility that CYP3A-mediated effects may modulate the overall efficacy in vivo; we therefore consider MEST a major effector, albeit not necessarily exclusive. A key limitation of this study is the absence of in vivo pharmacological dissection, as well as the lack of independent biophysical validation, including cellular thermal shift assay (CETSA) or isothermal titration calorimetry (ITC), to unequivocally confirm direct MEST binding and selectivity. Future investigations using CYP3A-deficient animal models, co-treatment with structurally distinct and MEST-sparing CYP3A inhibitors, and complementary biophysical binding assays will be essential to fully elucidate the mechanistic landscape and to guide the rational design of more selective MEST-targeting therapeutic agents.

In summary, we identified MEST as a key oncogenic driver that orchestrates GC initiation and progression via activation of the NF-κB signaling pathway. Cobicistat acts as a candidate MEST-inhibitory compound that suppresses tumor progression across multiple preclinical models, including patient-derived organoids and xenografts, by attenuating the MEST-NF-κB axis, highlighting its therapeutic efficacy. However, this study is restricted to preclinical validation without large-scale clinical confirmation, and further exploration is needed to clarify potential drug off-target risks and optimize clinical translation strategies. Therefore, cobicistat, as a potential therapeutic candidate targeting MEST, warrants further in-depth investigation.

## Data Availability

The original contributions presented in the study are included in the article/**Supplementary Material**. Further inquiries can be directed to the corresponding authors.
