# Supplementary material for: Targeting MEST using cobicistat as a therapeutic strategy for gastric cancer through suppressing NF-κB signaling
Source: Front Oncol. 2026 Aug 4;16:1901635. doi: 10.3389/fonc.2026.1901635 (PMC13481203; doi:10.3389/fonc.2026.1901635)
Supplement: Supplementary file 1 [file Table1.docx]

**Supplementary Material**

**1 Supplementary Methods**

**CRISPR-Cas9-Mediated gene knockout.** sgRNAs targeting MEST were designed and cloned into the lenti-CRISPR-Cas9 vector. Lentiviral particles were produced using a packaging plasmid system and used to transduce AGS and MKN45 cells. Successfully transduced cells were selected with puromycin, and knockout efficiency was confirmed by immunoblotting.

**Plasmid construction.** The target gene fragment was inserted into a plasmid vector. The resulting recombinant plasmids were transformed into competent E. coli cells. Positive clones were selected via antibiotic resistance, amplified, and subjected to plasmid extraction. The sequence of the inserted gene fragment was confirmed by Sanger sequencing.

**Lentiviral transduction.** A non-targeting control shRNA was purchased from GENECHEM. Tumor cells were transduced with lentiviral particles carrying shRNA in medium supplemented with 5 μg/mL polybrene for 24 h. Cells were subsequently selected and maintained in medium containing 2-4 μg/mL puromycin.

**Cell culture.** All GC cell lines, including MKN45 and AGS, were obtained from the Cell Bank of the Chinese Academy of Sciences. Cells were cultured in complete medium (DMEM or RPMI-1640) supplemented with 10% fetal bovine serum (FBS) and 1% penicillin-streptomycin. Cultures were maintained at 37 °C in a humidified incubator containing 5% CO₂. Cells were routinely tested to confirm the absence of mycoplasma contamination.

**Cell viability and colony formation assays.** For cell viability assays, 5 × 10³ cells were seeded per well in 96-well plates and allowed to adhere overnight at 37 °C. Cells were then treated with serial concentrations of cobicistat for 48 h. Cell viability was assessed using the Cell Counting Kit-8 (CCK-8) assay according to the manufacturer’s instructions. For patient-derived organoid (PDO) assays, approximately 100 spheroids per well were embedded in Basement Membrane Extract (BME) diluted in complete medium and seeded in 96-well plates. After 5 days of cobicistat treatment, PDO viability was measured using the CellTiter-Glo® 3D Cell Viability Assay Kit. The half-maximal inhibitory concentration (IC₅₀) values were calculated using GraphPad Prism 8.0 software. For colony formation assays, 1 × 10³ cells were seeded in 35-mm dishes. After 14 days of incubation, the resulting colonies were fixed with 4% formaldehyde, stained with 0.05% crystal violet, and counted using ImageJ software.

**Transwell invasion and wound healing assays.** For invasion assays, 2 × 10⁵ cells suspended in serum-free medium were seeded into the upper chamber of Matrigel-coated Transwell inserts. The lower chamber was filled with complete medium supplemented with 10% fetal bovine serum (FBS) as a chemoattractant. After 48 h of incubation, cells that had invaded through the Matrigel and migrated to the lower surface of the inserts were fixed with 4% (v/v) formaldehyde, stained with 0.05% (w/v) crystal violet, and counted under a light microscope. For wound healing (scratch) assays, 2 × 10⁵ cells were seeded per well in 6-well plates and grown to 90-100% confluency. A uniform scratch was created using a sterile 200-μL pipette tip. Non-adherent cells were removed by gentle washing with phosphate-buffered saline (PBS), and the cells were then cultured in serum-free medium. Wound closure was monitored and quantified at 0 and 48 h using ImageJ software.

**Immunoblotting.** Protein lysates from cultured cells or tumor tissues were separated by SDS-PAGE and electrotransferred onto polyvinylidene difluoride (PVDF) membranes. The membranes were incubated with the appropriate primary antibodies at 4 °C overnight, followed by incubation with horseradish peroxidase (HRP)-conjugated secondary antibodies. Immunoreactive signals were detected using a MiniChemi 610 Plus chemiluminescent imaging system. The following antibodies were used in this study: anti-MEST (1:1000, Bioswamp), anti-GAPDH (1:5000, Santa Cruz Biotechnology), and HRP-conjugated goat anti-mouse or anti-rabbit IgG (1:3000-1:8000, Servicebio).

**2 Supplementary figure**

**Figure S1. Related to Figure 5.** (A) The mRNA levels of MEST was detected by qPCR following treatment with different concentrations of cobicistat.

**3 Supplementary table**

**Table S1** Clinical characterisics of GC patients.

| **Clinical characterisicse** | Gastric cancer patient |
| --- | --- |
| Age（year）  Gender  Tumor markers  Neoadjuvant chemotherapy  Operation style  Operation time(min)  Tumor location  Tumor size  Lauren classification  TNM stage  Lmmunohistochemistry  Ki67  Postoperative Survival  （mouth） | 56  Male  AFP 5.31ng/ml  CA125 6.20U/ml  CA199 4.62U/ml  CA72-4 7.60U/ml  None  Laparoscopic subtotal gastrectomy  155min  Gastric antrum-body junction  3×2×0.5cm  diffuse type  T3N1M0  CK8/18(+)  CKp（+）  p53（+）  PMS-2(+)  MLH-1(+)  MSH-6(+)  MSH-2(+)  Syn（+）  70%  Alive |

**Table S2** Details of eight small molecule binding pockets of MEST.

| Site | Size | PLB | Hyd | Side | Residues |
| --- | --- | --- | --- | --- | --- |
| 1 | 49 | 2.92 | 17 | 33 | TYR28 LEU29 HIS30 ILE31 PRO32 PRO33 PRO34 THR205 ARG206 MET208 ASN209 PHE210 TRP236 ARG240 ASP243 ASN245 LEU246 |
| 2 | 42 | 2.55 | 13 | 22 | LEU269 ALA270 SER271 VAL272 THR273 ILE274 PRO275 ILE276 THR299 LEU300 PRO301 ARG302 SER303 THR304 |
| 3 | 68 | 1.62 | 34 | 53 | PHE78 PRO79 ASP147 HIS185 LEU188 LEU190 GLN191 LEU194 MET208 VAL212 PHE213 GLY216 LEU217 VAL220 ILE248 LEU252 TYR254 VAL286 HIS314 TYR315 |
| 4 | 14 | 1.17 | 11 | 21 | PRO275 ILE276 HIS277 ARG302 THR304 SER306 ALA327 GLY330 PHE331 SER334 PHE335 |
| 5 | 17 | 0.55 | 4 | 16 | LEU29 HIS30 ILE31 PRO32 PRO33 ARG240 ASN241 ASN242 ASP243 |
| 6 | 13 | 0.39 | 9 | 16 | GLU128 ALA129 LEU131 ARG132 GLN137 GLY166 ARG167 LEU168 |
| 7 | 9 | 0.2 | 6 | 12 | HIS116 HIS117 TYR118 SER250 GLN253 |
| 8 | 25 | 0.16 | 7 | 12 | ARG159 TYR160 ASN163 ARG164 SER165 GLY166 ARG167 LEU168 THR169 |

**Table S3** Signal Value of 36 compounds in the docking score.

| NO. | ID | Compound name | Signal Value（RU） |
| --- | --- | --- | --- |
| 1 | T0207 | Ranolazine dihydrochloride | -2.719454 |
| 2 | T0966 | Sennoside A | 6.266746 |
| 3 | T1073 | Dronedarone hydrochloride | -0.977241 |
| 4 | T1100 | Polymyxin B Sulfate | 3.992158 |
| 5 | T1665 | Daptomycin | 1.318123 |
| 6 | T2119 | Venetoclax | 0.206543 |
| 7 | T1459 | Cisatracurium besylate | 20.248731 |
| 8 | T1799 | Caspofungin Acetate | 0.173856 |
| 9 | T6245 | BAPTA-AM | 0.031419 |
| 10 | T3906 | Epimedin A | 41.588184 |
| 11 | T3903 | Angoroside C | 5.127214 |
| 12 | T3893 | Forsythoside B | 71.113214 |
| 13 | T6246 | Cobicistat | 31.784271 |
| 14 | T3794 | Pentagalloylglucose | 3.270786 |
| 15 | T6437 | Cefoperazone | 4.289543 |
| 16 | T5026 | Oxytocin | 48.361675 |
| 17 | T5440 | Dbet57 | 28.084563 |
| 18 | T5434 | ARV-825 | 24.464789 |
| 19 | T5730 | MALTOHEXAOSE | 93.798533 |
| 20 | T7624 | DAPTA | 11.558563 |
| 21 | T7158 | Ombitasvir | -2.144587 |
| 22 | T14298 | Rimiducid | 76.809505 |
| 23 | T7601L | Carbetocin acetate | -1.589820 |
| 24 | T16153 | MS4078 | 1.358242 |
| 25 | T13954 | UNC6852 | 46.057191 |
| 26 | T2354 | UNC 0631 | 16.506494 |
| 27 | T6881L | LTX315 acetate | -1.889799 |
| 28 | T22344 | Icatibant Acetate | 0.883649 |
| 29 | T2099 | ABT-737 | 1.412696 |
| 30 | T7608 | Bremelanotide Acetate | 24.013163 |
| 31 | TP1035 | Cyclic somatostatin | -0.968268 |
| 32 | TP1929L1 | Saralasin acetate(34273-10-4 free base) | -1.248778 |
| 33 | T7339 | Angiotensin (1-7) | 85.850919 |
| 34 | T1245 | Colistin sulfate | 6.786307 |
| 35 | T7423 | Diquafosol tetrasodium | 0.234855 |
| 36 | T7092 | NADPH tetrasodium salt | 40.901775 |

**Table S4** Univariate/multivariate Cox survival analysis

|  | Univariate | | Multivariate | |
| --- | --- | --- | --- | --- |
| Variables | HR (95%CI) | P | HR (95%CI) | P |
| IHCscoregroup |  |  |  |  |
| ≤150 | 1.00 (Reference) |  | 1.00 (Reference) |  |
| >150 | 1.91 (1.21 ~ 3.02) | 0.006 | 1.885 (1.04 ~ 3.414) | 0.037 |
| Sex |  |  |  |  |
| Male | 1.00 (Reference) |  |  |  |
| Female | 1.44 (0.76 ~ 2.73) | 0.268 |  |  |
| Differentation |  |  |  |  |
| Well-differentiated/moderately to well-differentiated | 1.00 (Reference) |  |  |  |
| Moderately to poorly differentiated | 0.93 (0.32 ~ 2.73) | 0.899 |  |  |
| Poorly differentiated | 1.78 (0.60 ~ 5.29) | 0.301 |  |  |
| Lauren |  |  |  |  |
| Intestinal-type | 1.00 (Reference) |  |  |  |
| Diffuse-type | 1.94 (0.98 ~ 3.85) | 0.059 |  |  |
| Mixed-type | 0.92 (0.39 ~ 2.16) | 0.840 |  |  |
| Location |  |  |  |  |
| Upper 1/3 | 1.00 (Reference) |  |  |  |
| Middle 1/3 | 0.82 (0.37 ~ 1.79) | 0.614 |  |  |
| Lower 1/3 | 0.85 (0.42 ~ 1.71) | 0.642 |  |  |
| Age | 0.98 (0.96 ~ 1.01) | 0.277 |  |  |

**Table S5** Clinicopathological characteristics in gastric cancer patient**s.**

| Characteristics | Number | MEST expression | P-value |
| --- | --- | --- | --- |
| Gender |  |  |  |
| Female | 35 | 149.1 ± 57.5 | 0.009 |
| Male | 130 | 118.5 ± 68.9 |  |
| Age (years) |  |  |  |
| <60 | 82 | 121.3 ± 62.3 | 0.495 |
| ≥60 | 83 | 128.6 ± 72.7 |  |
| Lauren classification |  |  |  |
| Intestinal | 60 | 121.5 ± 66.5 | 0.622 |
| Diffuse | 46 | 120.7 ± 67.1 |  |
| Mixed | 59 | 131.9 ± 69.7 |  |
| TNM stage |  |  |  |
| I + II | 56 | 125.4 ± 62.0 | 0.956 |
| III + IV | 109 | 124.8 ± 70.6 |  |
| Differentiation |  |  |  |
| Well/moderate | 77 | 124.2 ± 63.0 | 0.885 |
| Poor | 88 | 125.7 ± 71.8 |  |
